# Adhesive Restorative Procedures for Anterior Esthetic Rehabilitation: A Case Series

**DOI:** 10.7759/cureus.109582

**Published:** 2026-05-25

**Authors:** Sachin Gupta, Shikha Jaiswal, Ankita Kumari, Gabbi Vimalpreet Kaur, Ridhiman Raman, Manasvi Sharma

**Affiliations:** 1 Department of Conservative Dentistry and Endodontics, Subharti Dental College and Hospital, Swami Vivekanand Subharti University, Meerut, IND

**Keywords:** adhesive dentistry, anterior esthetics, composite resin, composite veneers, direct composite veneering, esthetic buildup, fiber post, malalignment

## Abstract

The demand for esthetically driven dental treatment has increased substantially with advances in adhesive dentistry. This case series describes three anterior esthetic rehabilitation scenarios: (i) direct composite veneering for correction of anterior malalignment in vital teeth, (ii) combined in-office bleaching and direct composite veneering for fluorosis-associated discoloration in vital teeth, and (iii) conservative rehabilitation of structurally compromised endodontically treated teeth with sodium ascorbate neutralization, and anatomic fiber post fabrication with adhesive core reconstruction. All cases demonstrated satisfactory immediate esthetic and functional outcomes. Follow-up evaluations showed stability of the restorations. Adhesive restorative procedures provide versatile and cost-effective solutions for anterior esthetic rehabilitation across a broad spectrum of clinical presentations. When executed with appropriate case selection, meticulous adhesive protocols, and systematic finishing and polishing, direct composite restorations and adhesive post-endodontic rehabilitation can provide predictable esthetic and functional outcomes.

## Introduction

The restoration and re-establishment of dental esthetics are fundamental objectives of contemporary restorative dentistry [1]. With increasing patient awareness and demand for immediate esthetic enhancement, clinicians are expected to deliver biologically respectful and time-efficient restorative solutions [2]. Advances in adhesive dentistry have broadened the scope of esthetic restorative procedures, enabling more conservative, predictable, and tooth-preserving treatment modalities [3].

Among available esthetic options, porcelain veneers remain widely regarded as the gold standard when appropriately indicated because of their optical properties, surface gloss, and long-term color stability. However, laboratory-fabricated ceramic veneers are technique-sensitive, relatively costly, and often require irreversible enamel reduction. These limitations have stimulated the evolution of direct composite alternatives that align with the principles of conservative dentistry [4].

The development of advanced microhybrid and nanohybrid composite resin systems has positioned direct composite veneering as a highly viable conservative alternative. Contemporary composites demonstrate improved mechanical strength, wear resistance, polish retention, and optical blending that closely simulate porcelain esthetics [5]. Direct composite veneering involves the incremental layering and sculpting of resin material directly onto the tooth surface to correct color, contour, alignment, and morphology [4].

Beyond conventional esthetic indications such as discoloration, diastema closure, and mild malalignment in non-endodontically treated teeth, adhesive composite techniques have expanded to address more complex restorative challenges. These include the immediate rehabilitation of endodontically treated teeth with structurally compromised and flared canals, where reduced radicular dentin thickness may preclude predictable use of conventional prefabricated post systems. In such situations, the anatomic post technique, wherein a fiber post is relined with composite resin to conform to irregular canal geometry, provides improved adaptation, reduced cement film thickness, improved stress distribution, and enhanced biomechanical stability of the remaining tooth structure [6]. This approach offers an effective, tissue-preserving alternative to traditional cast post and core systems [6,7].

In patients seeking rapid esthetic improvement, adhesive restorative techniques may also be combined with adjunctive procedures, such as in-office bleaching. In fluorosis-associated discoloration, bleaching can reduce intrinsic stain intensity, while direct composite veneering allows correction of residual discoloration, contour irregularities, and mild malalignment. Where same-visit bleaching and bonding are performed, antioxidant neutralization with sodium ascorbate is necessary to counter residual oxygen radicals and support predictable adhesive bonding [8,9].

This case series presents three clinical scenarios: (i) direct composite veneering for anterior malalignment correction in vital teeth, (ii) combined bleaching and direct composite veneering for fluorosis-associated discoloration in vital teeth, and (iii) conservative adhesive rehabilitation of endodontically treated teeth with flared canals using anatomically customized fiber posts.

## Case presentation

Informed consent was obtained from all patients after explaining the proposed procedures, alternative treatment options, possible risks, benefits, and expected prognosis. A digital diagnostic mock-up was performed using InLab CAD Software version 22.0 (Dentsply Sirona Inc., Charlotte, North Carolina, United States) for all three cases to visualize the proposed esthetic outcome, guide treatment planning, and enhance patient education and consent.

Case 1: direct composite veneering for anterior malalignment correction

A 23-year-old male patient presented with a fractured anterior tooth associated with malalignment, resulting in compromised esthetics. Clinical examination revealed an incisal fracture of vital tooth 11 with mild midline discrepancy and disharmony in anterior alignment.

**Figure 1:**
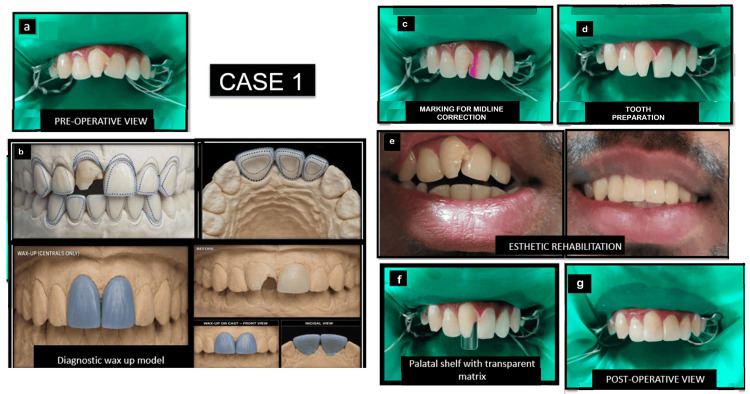
Direct composite veneering for anterior malalignment correction (Case 1)

Preoperative assessment was performed using labial, profile, and incisal photographic views to evaluate proportions, symmetry, and occlusion( Figure 1a). Treatment options, including orthodontic correction and indirect restorations, were discussed. However, owing to financial constraints, time limitations, immediate esthetic demands, and the need to preserve tooth structure, a direct composite approach was selected.

Direct clinical measurements were performed using a digital caliper and periodontal probe to quantify the extent of midline deviation in relation to the facial midline (glabella-philtrum-chin reference) and dental midline. A digital diagnostic wax-up model was created using InLab CAD software (Figure 1b). The width-to-length ratios of the maxillary central incisors and the amount of mesiodistal discrepancy were assessed to determine the extent of additive versus subtractive correction required. A diagnostic mock-up and reference markings were used to estimate the minimal enamel reduction necessary to correct the midline shift while maintaining proportional harmony (Figure 1c). Diagnostic markings were performed to guide midline correction and contour modification proximally and incisally with respect to tooth 21, ensuring an additive strategy with no unnecessary tooth preparation (Figure 1d). Subsequently, minimal tooth preparation along with proximal shelf preparation was carried out with respect to tooth 11 to receive a direct composite veneer. Final esthetic rehabilitation was accomplished using a direct composite restorative technique (Figure 1e).

Enamel surfaces were etched with 37% phosphoric acid (3M™ Scotchbond™ Multi-Purpose Etchant 3007; 3M Company, Maplewood, Minnesota, United States). The composite resin was carefully adapted to the prepared surfaces and polymerized using a light-curing unit. The composite resin restoration (3M™ Filtek™ Ultimate Universal Restorative; 3M Company) was built incrementally, employing a transparent matrix as a template to achieve accurate contour and proximal form (Figure 1f). Occlusal contacts were evaluated and adjusted accordingly.

Postoperative evaluation demonstrated satisfactory correction of the midline, restoration of the fractured tooth, and improved alignment and symmetry of the anterior segment. The direct composite restorations blended harmoniously with adjacent teeth, providing optimal esthetics and function while adhering to the principles of minimal intervention dentistry. (Figure 1g).

Case 2: combined bleaching and direct composite veneering for fluorosis-associated discoloration

A 20-year-old female patient presented with discolored and malaligned maxillary anterior teeth. The patient had no significant medical or dental history. Clinical examination revealed a Thylstrup-Fejerskov (TF) Index Score 7 enamel fluorosis of the maxillary anterior dentition, with crowding and lingual displacement of the central and lateral incisors.

**Figure 2:**
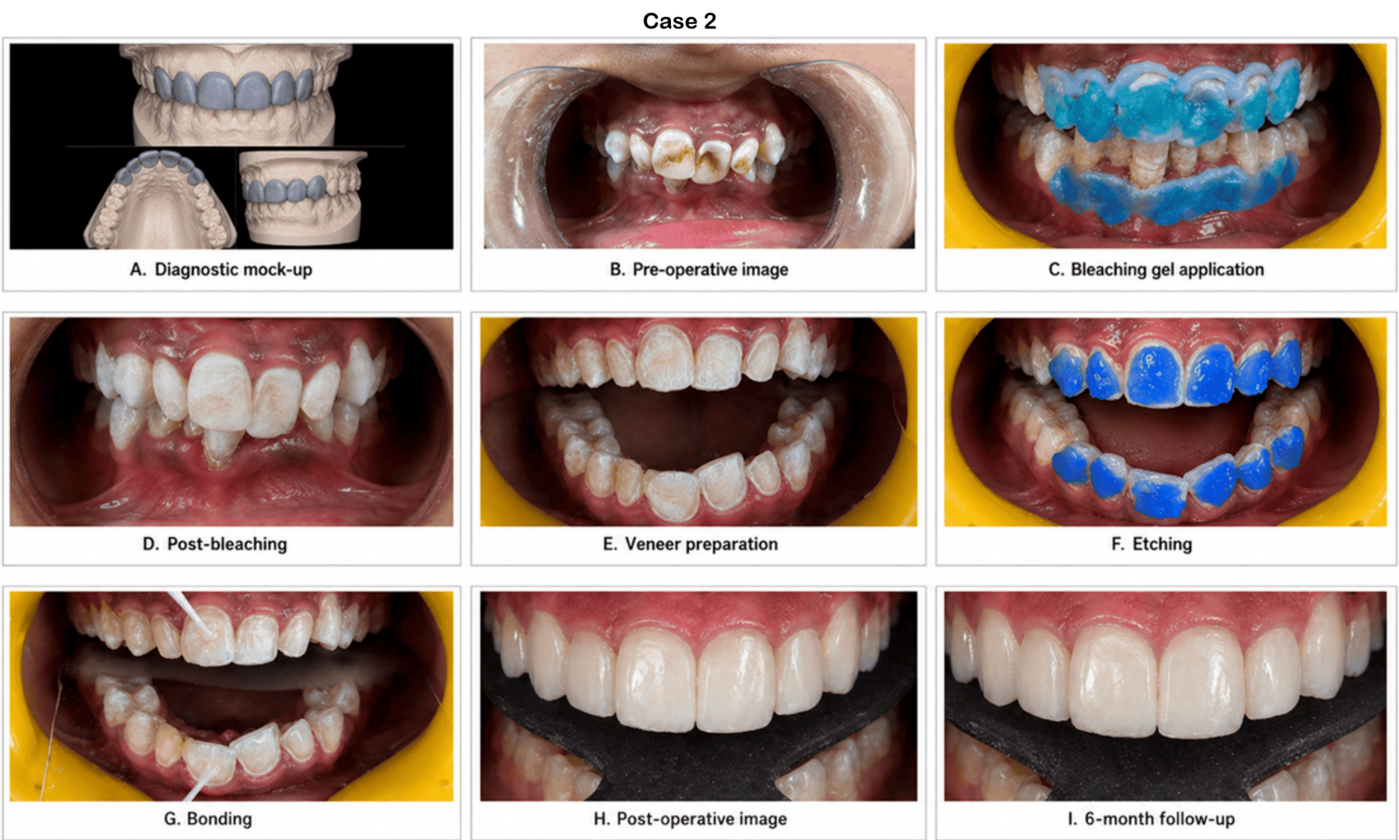
Combined bleaching and direct composite veneering for fluorosis-associated discoloration (Case 2)

An interdisciplinary treatment plan including orthodontic alignment, discoloration management, and definitive composite restorations was proposed. However, because the patient was unwilling to undergo orthodontic treatment and required immediate esthetic rehabilitation before an impending family function, a single-visit approach comprising in-office bleaching followed by direct composite veneering was selected as a pragmatic, evidence-based approach. Prior to treatment, a digital diagnostic mock-up was performed using InLab CAD software (Figure 2A).

Management of Tooth Discoloration

Following oral prophylaxis, the preoperative shade was recorded as C4 using the Vita Classical Shade Guide (ITA Zahnfabrik H. Rauter GmbH & Co. KG, Bad Säckingen, Germany), and baseline photographs were obtained (Figure 2B). After gingival barrier placement, in-office bleaching was performed using Pola Office gel (SDI Limited, Victoria, Australia), activated for three consecutive eight-minute light activation cycles according to the manufacturer’s protocol (Figure 2C).

Composite bonding was planned for the same visit to meet the patient’s immediate esthetic need. However, hydrogen peroxide-based bleaching agents generate residual oxygen radicals that impair resin polymerization at the adhesive interface and reduce bond strength when bonding is performed immediately after bleaching [8]. To neutralize this effect, 25% sodium ascorbate solution was applied to the bleached enamel surfaces for five minutes prior to bonding as recommended by Kaya et al. [8]. Post-bleaching shade improvement is illustrated in Figure 2D.

Minimal-Preparation Direct Composite Veneer

Shade selection was performed under standardized lighting. Enamel recontouring was performed from canine to canine. Labial preparation of 0.3-0.5 mm depth was carried out using depth-cutting burs from the Shofu Porcelain Veneer Kit (Shofu Dental, Kyoto, Japan) (Figure 2E). Enamel was etched with 37% phosphoric acid (KaVo Dental, Biberach, Germany) for 20 seconds, rinsed, and gently air dried (Figure 2F). A bonding agent was applied and light-cured for 20 seconds (Figure 2G). Nanohybrid composite was placed incrementally to restore morphology and esthetics, achieving an immediate post-operative improvement (Figure 2H). Follow-up evaluations at six months demonstrated excellent marginal adaptation, satisfactory shade blending, and surface gloss retention, with selective repolishing performed at the six-month recall (Figure 2I).

Case 3: conservative rehabilitation of endodontically treated teeth with flared canals using anatomically customized fiber posts

A 19-year-old male patient presented with fractured maxillary anterior teeth that had undergone previous root canal treatment (two years ago). The fracture occurred a day before presentation in the root canal-treated teeth, which had not been covered by a crown, and the patient desired immediate rehabilitation due to an upcoming family function.

**Figure 3:**
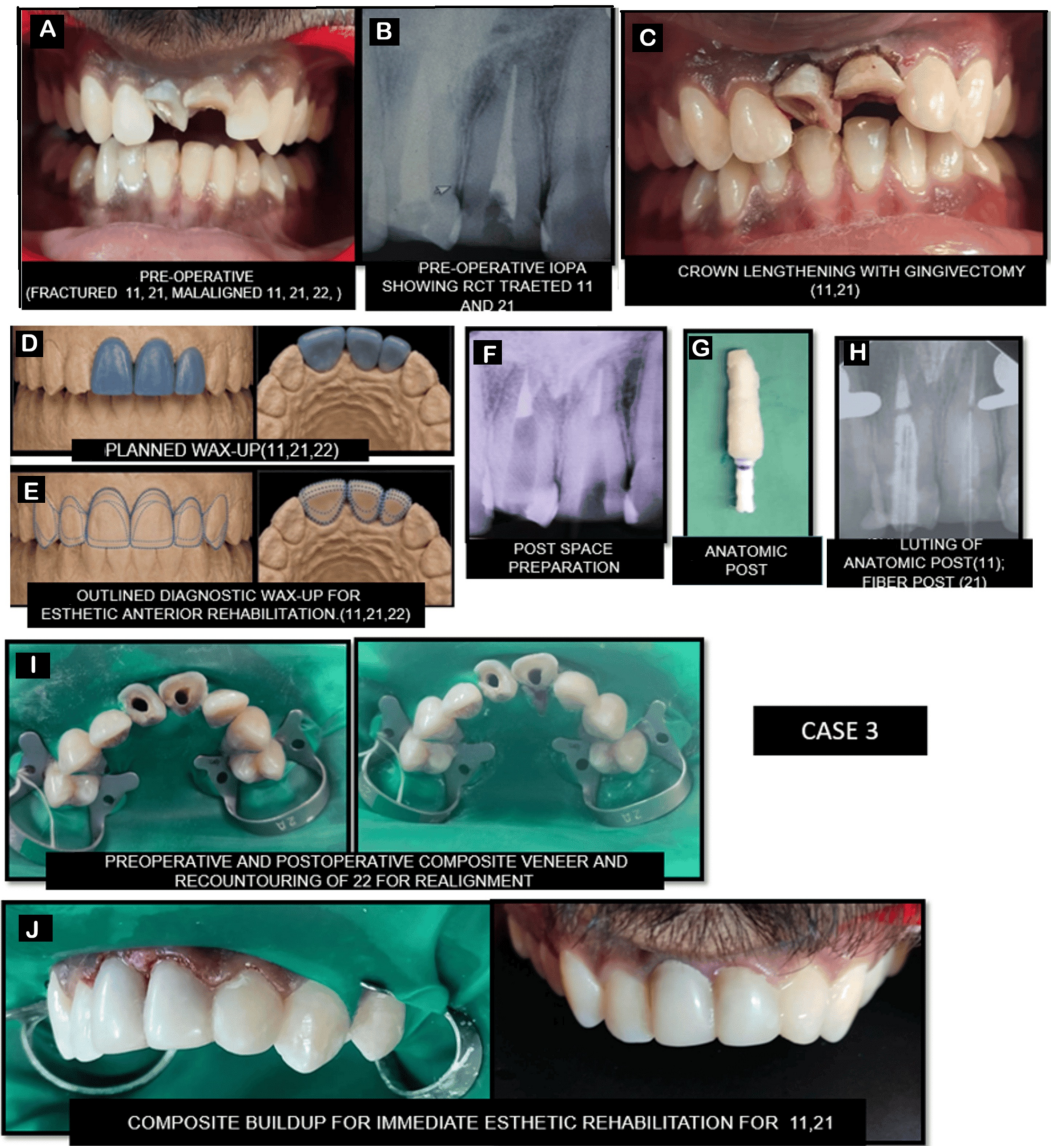
Conservative rehabilitation of endodontically treated teeth with flared canals using anatomically customized fiber posts (Case 3)

Clinical examination revealed fractured and rotated 11 and 21 with mild rotation in vital 22. The patient was asymptomatic, with no pain, swelling, tenderness, or mobility in 11 and 21 (Figure 3A).

Radiographic examination showed root canal-treated 11 and 21 with satisfactory obturation and with no periodontal ligament widening or periapical pathology present. Additionally, 11 showed flared root canals with reduced dentin thickness, while tooth 21 had regular canal anatomy (Figure 3B).

Due to the need for immediate rehabilitation, a customized anatomic fiber post was planned in 11 (due to its flared canal anatomy and reduced dentinal thickness), while tooth 21 was planned for a prefabricated fiber post along with a complete composite buildup of 11 and 21 with simultaneous correction of rotated 21 after performing gingivectomy using electro cautery (to increase the axial crown height for better esthetics and better retention from intact residual enamel) (Figure 3C). A digital diagnostic wax-up model was created with InLab CAD software (Figures 3D, 3E).

A prefabricated glass fiber post number 2 was selected for 21, while an anatomic post technique was used for 11. Gutta-percha was initially removed using heated pluggers followed by post space enlargement in 21 using Peeso reamers (Mani, Inc., Utsunomiya, Japan) up to size No. 3, whereas no preparation was needed in 11 (Figure 3F).

For Anatomic post fabrication, a number 2 prefabricated glass fiber post (Angelus Reforpost; Angelus Indústria de Produtos Odontológicos S/A, Londrina, Brazil) was relined with composite. The post was cleaned with ethyl alcohol, treated with silane (Silano; Angelus Indústria de Produtos Odontológicos S/A) for 60 seconds, air dried, and coated with Scotchbond Universal Adhesive (3M Company). Tetric N-Ceram composite shade A2 (Ivoclar Vivadent AG, Schaan, Liechtenstein) was adapted around the post and inserted into the lubricated post space (Figure 3G).

The post space was lightly lubricated with glycerin to prevent adhesion of the composite to canal walls and allow easy post removal. After initial intra-canal light curing, the anatomic post was removed, light cured extraorally for 40 seconds, finished, and checked for passive fit. The lubricant was cleaned from the post space, followed by luting of the anatomic post.

The post spaces in 11 and 21 were coated with Scotchbond Universal Adhesive (3M Company) as per protocol. Dual-cure resin cement (RelyX; 3M Company) was delivered using an elongation tip to ensure proper placement and minimize air entrapment. The anatomic post and fibre posts were seated with gentle pressure, excess cement removed, and light cured for 40 seconds from multiple directions (Figure 3H).

Immediate esthetic correction was done by building up the entire crown by incremental placement of composite using Tetric N-Ceram composite shade A2 (Ivoclar Vivadent AG). During build-up, mild rotation in tooth 11, 22 was corrected by modifying the mesial contour, enhancing labial prominence, and refining line angles to improve immediate esthetic alignment (Figures 3I, 3J).

Finishing and Polishing: Common Protocol for All Cases

Initial contouring was accomplished with aluminum oxide discs, 3M™ Sof-Lex™ Pop-on Contouring and Polishing Discs (3M Company), and carbide finishing burs (FG 7714F, FG 9714FF). Interproximal excess was removed with a No. 12 scalpel blade, while palatal surfaces were adjusted with fine diamond points (3118, 3118F).

Final polishing was performed using Sof-Lex discs, polishing rubbers, and silicon carbide-impregnated brushes (Astrobrush; Ivoclar Vivadent AG). Aluminum oxide polishing paste (Enamelize; Cosmedent, Inc., Chicago, Illinois, United States) was applied to achieve surface gloss and enamel-like smoothness [10,11].

Follow-ups

 At three to six-month follow-ups, the restorations remained functional and esthetically stable, with satisfactory marginal integrity, shade match, surface gloss, and no postoperative sensitivity, marginal staining, or chipping.

Patient perspectives

The patients expressed high satisfaction with the immediate esthetic improvement and appreciated the conservative, single-visit nature of the procedure.

## Discussion

Esthetic rehabilitation of anterior teeth is a primary objective of contemporary restorative dentistry, where treatment success depends not only on functional recovery but also on restoration of a natural appearance. The three cases presented here collectively illustrate the clinical scenarios in which adhesive restorative procedures can serve as conservative, esthetic and functional treatment modalities for immediate rehabilitation.

An important adjunct common to all cases was the use of a digital diagnostic mock-up, which played a pivotal role in enhancing treatment predictability and execution. Digital mock-ups allow preoperative visualization of the proposed esthetic outcome, enabling precise assessment of tooth proportions, symmetry, contour, and smile harmony without irreversible intervention. This facilitates an outcome-driven approach to treatment planning while minimizing unnecessary tooth preparation. Furthermore, the visual simulation significantly improves patient communication and acceptance by aligning patient expectations with achievable clinical results. From a procedural standpoint, the mock-up serves as a practical reference during restorative execution, guiding contouring, layering, and midline correction. Thus, its incorporation contributes to improved esthetic predictability, procedural efficiency, and overall clinical confidence in adhesive restorative workflows [12,13].

Porcelain veneers have long been considered the gold standard for anterior esthetic rehabilitation because of their optical depth, surface gloss, and long-term color stability. Nevertheless, their fabrication requires laboratory support, involves at least two clinical visits and demands irreversible enamel reduction, making them unsuitable when single-visit treatment, tooth structure preservation, or cost containment is a priority [4]. In contrast, direct composite veneering achieves esthetically acceptable outcomes in a single appointment with minimal or no preparation, preserving maximum enamel for future intervention.

Contemporary nanohybrid composite systems demonstrate improved mechanical strength, wear resistance, polish retention and optical properties that closely approximate natural enamel and dentin [4]. Studies confirm that direct composite veneers exhibit excellent esthetic integration and satisfactory long-term performance when placed with proper adhesive protocols and multi-step finishing [10]. In young patients, such as those in Cases 1 and 2, the reversibility and reparability of direct composite restorations represent a decisive clinical advantage over irreversible ceramic preparations [4].

The predictability of direct composite restorations is fundamentally contingent on the quality of the adhesive interface. In the composite veneering cases, enamel was etched with 37% phosphoric acid for 20 seconds followed by a universal adhesive, a protocol consistent with the established evidence base for enamel bonding. Phosphoric acid selectively demineralizes enamel prisms, creating microporosities into which adhesive resin penetrates to form resin tags and achieve micromechanical retention. Universal adhesives additionally provide chemical adhesion through functional monomers such as 10-MDP, which bonds ionically to hydroxyapatite [7].

In Case 3, the adhesive challenge extended to the post-cement-dentin interface at depth. Dual-cure resin cement was selected to ensure adequate polymerization within the canal, where light access is limited. The combination of chemical and light activation ensures consistent conversion throughout the cement layer, optimizing bond strength and minimizing microleakage [14]. Scotchbond Universal was applied to canal walls prior to cement delivery to support bonding to radicular dentin.

The management of fluorosis-associated discoloration in Case 2 illustrates the effectiveness of combined bleaching and direct composite veneering for rapid esthetic improvement. Bleaching with hydrogen peroxide reduces intrinsic stain severity, minimizes the composite volume required, and enhances optical blending with adjacent natural dentition [13]. The critical clinical decision in this case was the application of 25% sodium ascorbate solution prior to bonding.

Hydrogen peroxide bleaching agents generate free oxygen radicals that inhibit resin polymerization at the adhesive interface, substantially reducing bond strength when bonding is performed immediately after bleaching [13]. Sodium ascorbate scavenges these radicals and restores the oxidation-reduction potential of bleached enamel to baseline. Kaya et al. demonstrated that 25% sodium ascorbate applied for five minutes was sufficient to restore shear bond strength values comparable to unbleached enamel [8], making this an evidence-based protocol step for same-visit bleaching and bonding when clinical circumstances require immediate treatment.

Endodontically treated teeth with flared canals pose a particularly demanding restorative challenge. Conventional prefabricated posts of standard dimensions cannot conform to irregularly shaped large canals, resulting in a wide, non-uniform cement annulus that concentrates stress at the post-dentin interface and elevates the risk of root fracture [14]. The anatomic post technique, in which a fiber post is relined with composite to produce a custom-fit assembly, minimizes this limitation. By reducing the thickness of the cement layer, this approach decreases polymerization shrinkage stress, improves load transfer along the root length, and promotes improved stress distribution and enhanced biomechanical stability of the remaining tooth structure [15].

The anatomic post technique represents a fundamentally conservative approach to the rehabilitation of flared canals. Unlike traditional protocols that may necessitate aggressive radicular preparation to accommodate a larger prefabricated post, this technique prioritizes the preservation of residual dentin. By adapting the restorative material to the existing canal morphology, clinicians can maintain the structural integrity of the root, which is a critical factor in the long-term prognosis of endodontically treated teeth [14,15].

The use of glass fiber posts confers further biomechanical advantages. Their modulus of elasticity approximates that of dentin, producing a more homogeneous stress distribution throughout the restored tooth compared with rigid metallic posts [6]. Studies demonstrate that anatomically adapted fiber posts exhibit superior retention and fracture resistance in wide canals relative to conventional prefabricated posts while preserving dentin thickness that would otherwise be sacrificed by aggressive post-space enlargement [14]. In Case 3, this strategy enabled conservative rehabilitation without unnecessary radicular dentin removal, directly supporting the philosophy of this manuscript.

Finishing and polishing protocols play a critical role in the longevity and esthetic success of composite restorations. A well-polished surface reduces plaque accumulation, improves color stability, and enhances gloss and natural appearance. Multi-step polishing with aluminum oxide discs and polishing pastes produces enamel-like surface smoothness and long-term gloss retention [10,11]. In all three cases, a systematic multi-step finishing protocol ensured that the final restorations exhibited surface texture and optical properties consistent with the surrounding dentition.

## Conclusions

This case series demonstrates that adhesive restorative procedures offer a versatile, conservative, and cost-effective approach for anterior esthetic rehabilitation across a spectrum of clinical presentations, including malalignment correction in a non-endodontically treated tooth, fluorosis-associated discoloration management in intact dentition, and conservative post-endodontic rehabilitation of structurally compromised teeth with flared canals.

Direct composite veneering provided immediate esthetic correction while preserving tooth structure in Cases 1 and 2. In Case 2, the incorporation of sodium ascorbate neutralization prior to same-visit bonding after in-office bleaching served as an evidence-based protocol step to support predictable adhesion within the constraints of an urgent clinical timeline. In Case 3, the strategic use of anatomically customized fiber posts enabled sound rehabilitation while preserving residual radicular dentin, which is particularly important in young patients where long-term tooth preservation is paramount.
